# Effect of probiotics derivatives (postbiotics and metabiotics) on glycemic control in individuals with type 2 diabetes: a randomized clinical trial (DELI_Diab study)

**DOI:** 10.3389/fendo.2026.1802045

**Published:** 2026-05-04

**Authors:** Maryana Savytska, Olena Baka, Elina Manzhalii, Dmytro Kyriienko, Tetyana Falalyeyeva, Olena Strubchevska, Serhii Futornyi, Viktoriia Pastukhova, Halyna Lukyantseva, Liubov Sichel, Nazarii Kobyliak

**Affiliations:** 1 Danylo Halytsky Lviv National Medical University, Lviv, Ukraine; 2 Center for Innovative Medical Technologies of the National Academy of Sciences of Ukraine, Kyiv, Ukraine; 3 Bogomolets National Medical University, Kyiv, Ukraine; 4 Kyiv City Clinical Endocrinology Center, Kyiv, Ukraine; 5 Taras Shevchenko National University of Kyiv, Kyiv, Ukraine; 6 Medical Laboratory CSD, Scientific, Kyiv, Ukraine; 7 Jagiellonian University Medical College, Krakow, Poland; 8 National University of Ukraine on Physical Education and Sport, Kyiv, Ukraine; 9 MirImmunoFarm, Kyiv, Ukraine

**Keywords:** glycemic control, gut microbiota, insulin resistance, metabiotics, postbiotics, probiotic derivatives, type 2 diabetes, β-cell dysfunction

## Abstract

**Introduction:**

Probiotics have demonstrated positive effects on obesity and type 2 diabetes (T2D) in various studies. Postbiotics, specifically nonliving bacterial lysates derived from cell walls and DNA, are increasingly recognized as safe, stable, and effective alternatives for improving host metabolic health.

**Aim:**

to conduct a randomized, double-blind, placebo-controlled clinical trial (RCT) to assess the short-term safety and effectiveness of the bacterial lysate of *Lactobacillus (L.) rhamnosus* DV - NRRL B-68023 as an adjunct to standard antidiabetic therapy.

**Material and methods:**

Fifty-five participants with T2D were enrolled and assigned via computer-generated randomization to receive either 100 mg of *L. rhamnosus DV* lysate twice daily or a matching placebo. Blinding was ensured through identical masked packaging. The primary efficacy analysis was performed on a per-protocol (PP) population of 42 participants (intervention, n=20; placebo, n=22) who completed the 3-month treatment and 3-month follow-up. Primary endpoints were HbA1c and fasting plasma glucose (FPG). Secondary endpoints included HOMA-2 modeled insulin sensitivity (%S), β-cell function (%B), and anthropometric parameters, evaluated at baseline, 3, and 6 months. The trial was registered at ClinicalTrials.gov (NCT05770076).

**Results:**

The primary analysis revealed a significant overall treatment effect for HbA1c (p<0.001). After 3 months, the intervention group showed a mean HbA1c reduction of –0.63% (95% CI: –0.98 to –0.28), while the placebo group significantly worsened (0.50%; 95% CI: 0.17 to 0.84). For FPG, a favorable between-group trend was observed (p=0.086), with a reduction of 1.32 mmol/L in the active group compared to an increase of 0.12 mmol/L in the placebo group. Exploratory analysis showed significant treatment effects for waist circumference (p = 0.021) and insulin sensitivity (%S, p = 0.013). Although within-group improvements were noted for BMI and β-cell function (%B) in the intervention group (p<0.05), between-group differences for these parameters were not statistically significant (p>0.05).

**Conclusions:**

Three-month supplementation with *L. rhamnosus DV* lysate significantly improves glycemic control (HbA1c) and reduces waist circumference compared to placebo in T2D patients. While positive trends in insulin sensitivity and anthropometrics were observed, further large-scale studies are required to confirm these metabolic benefits and assess long-term sustainability.

**Clinical Trial Registration:**

https://clinicaltrials.gov/keywords, identifier NCT05770076.

## Introduction

Type 2 diabetes (T2D) is a complex metabolic disorder marked by insulin resistance, progressive dysfunction of pancreatic β-cells, and ongoing low-grade inflammation (1). Growing evidence suggests that changes in the composition and function of the gut microbiota may contribute to the metabolic dysregulation associated with the development of T2D (2–5). This influence can occur through immune activation, impairment of the intestinal barrier, and the modulation of host metabolism (4, 6).

Probiotics have been investigated as adjunctive strategies for improving glycaemic control in individuals with T2D. Several randomized clinical trials (RCT) and meta-analyses have demonstrated modest improvements in glycated hemoglobin (HbA1c), fasting plasma glucose (FPG), and inflammatory markers following probiotic supplementation (7–13). However, the magnitude and consistency of these effects vary substantially depending on strain specificity, treatment duration, baseline metabolic status, and study design (7). In addition, concerns regarding the stability, viability, and safety of live microorganisms in vulnerable populations (14–20) have stimulated interest in alternative microbiota-derived therapeutic approaches.

In this context, increasing attention has been directed toward non-viable microbial preparations and their biologically active structural components, collectively referred to as postbiotics and metabiotics (21). According to the International Scientific Association of Probiotics and Prebiotics (ISAPP), postbiotics are preparations of inanimate microorganisms and/or their components that confer health benefits to the host. Additional related terms have also been used, including ‘metabiotics’ (22, 23) and ‘bacterial lysates’ (23, 24). The concept that non-living microorganisms could promote or preserve health is not new, and several terms have been used to describe such substances, although postbiotic has been used most often in the past decade (23). Postbiotics containing cell wall fragments and nucleic acid components represent one example of such preparations and may exert immunomodulatory and metabolic effects without the need for viable bacterial colonization (25, 26).

Experimental and clinical studies suggest that bacterial lysates and postbiotic preparations can influence inflammatory signalling pathways and host metabolic regulation (27–31). However, randomized controlled trials evaluating their metabolic effects in individuals with T2D remain limited (32).

Therefore, the present double-blind, placebo-controlled RCT aimed to evaluate the short-term efficacy and safety of a lyophilized enzymatic bacterial lysate derived from *Lactobacillus (L.) rhamnosus* DV - NRRLB-68023 as an adjunct to standard antidiabetic therapy to improve glycaemic control, insulin sensitivity, β-cell functional activity, and anthropometric parameters in individuals with T2D.

## Materials and methods

### Ethics statement

This double-blind, placebo-controlled, parallel-group RCT was conducted in the Kyiv City Clinical Endocrinology Centre (Ukraine), with recruitment of patients beginning in March 2023. In compliance with the Declaration of Helsinki (1975), the study was implemented after obtaining approval from the local Ethics Committee (protocol 2/2023). The study has been registered in the ClinicalTrials.gov database (№ NCT05770076 DELI_Diab Study, registration date 03.03.2023). Before the initiation of the RCT, the study’s objectives and methods were clearly explained to the participants, and all patients voluntarily provided informed consent.

### Inclusion criteria

The criteria for inclusion in the study were as follows: participants aged 18–70 years with a body mass index (BMI) of 25–40 kg/m^2;^ and HbA1c levels between 6.5% and 10.0%. All individuals with a confirmed diagnosis of T2D, based on the American Diabetes Association guidelines, received either lifestyle therapy (diet and physical activity) or had been on a stable regimen including metformin, sulfonylureas (SUs), or insulin in stable dosage for a minimum of three months prior to enrollment, with daily insulin doses not exceeding 60 IU.

### Exclusion criteria

Participants were excluded from the study based on the following conditions: diagnosis of type 1 diabetes; presence of serious diabetes-associated complications at the time of screening, such as end-stage diabetic nephropathy, neuropathic pain requiring medication, proliferative retinopathy, or autonomic neuropathy. Also excluded were individuals using glucose-lowering therapies not listed in the inclusion criteria (e.g., pioglitazone, SGLT-2 inhibitors, GLP-1 receptor agonists, DPP-4 inhibitors). Additional exclusion factors included recent (within the past 3 months) use of antibiotics or microbiota-modulating supplements (such as pro-, pre-, post-, or synbiotics), known hypersensitivity to probiotics or their ingredients, and a history of gastrointestinal disorders, including food allergies, celiac disease, or nonspecific ulcerative colitis. Individuals with uncontrolled cardiovascular or respiratory conditions, decompensated liver disease (e.g., ascites, encephalopathy, variceal bleeding), active cancer or chronic infections were also excluded. Furthermore, participants who had experienced a severe form of COVID-19 requiring mechanical ventilation or ECMO, or who had a confirmed COVID-19 infection within 4 weeks prior to screening, were not eligible. Other exclusion criteria included concurrent participation in other clinical trials and pregnancy or breastfeeding.

### Study design

Fifty-five individuals with T2D who were taking oral hypoglycemic agents or minimal-dose insulin met the study’s inclusion criteria. The study included a screening period of up to one week to assess eligibility on the basis of inclusion/exclusion criteria, followed by treatment and follow-up periods, each lasting three months.

A preliminary period was implemented to minimize the impact of dietary changes on metabolic markers. This involved providing all patients with a single session with a dietician one week before randomization to make necessary lifestyle modifications. The nutrition program included a balanced diet and a recommended daily water intake of 30–40 ml/kg. The diet followed general balanced dietary recommendations for type 2 diabetes, based on the 2023 ADA guidelines (33) and the Harvard Healthy Eating Plate (34). The balanced diet was not tailored to individual caloric needs. Patients were given a list of approved and prohibited foods. Three meals a day with snacks were recommended. All cooking methods were acceptable except frying. The last meal of the day was consumed 1.5–2 hours before bedtime. Additionally, participants were encouraged to maintain their regular anti-diabetic medication regimen. None of the participants reported any changes in their medication type or dosage during the RCT. Dietary compliance was monitored by reviewing a food diary during follow-up visits, where a researcher cross-checked reported intake against the provided list of approved foods. High adherence to dietary recommendations defined as following the prescribed meal pattern >80% of the time.

The American Diabetes Association advocates for walking as a beneficial activity for patients with T2D (35). Participants were instructed to engage in light to moderate walking most days of the week, ideally aiming for daily activity. Some studies and walking-based activity guidelines suggest that achieving 5,000 to 8,000 steps can enhance well-being for individuals with T2D, as well as contribute to overall health maintenance for the general population (36–40). So in our RCT were advised to target a daily goal of 5,000 to 8,000 steps, with physical activity monitored using a pedometer. Activity levels were tracked using a pedometer and reviewed during follow-up visits.

To ensure compliance with the RCT, patients were required to maintain a diary documenting their supplementation, diet, and exercise. In this diary, they also recorded any AEs. Patients were encouraged to report and discuss any significant dietary changes during each follow-up visit. Any participant who reported major deviations from the baseline lifestyle or medication regimen was planned to be excluded from the per-protocol analysis to minimize confounding.

The RCT involved patients with T2D who were randomly assigned in a 1:1 ratio to receive either “Del-Immune V^®^ Extra” or a placebo for 3 months. To ensure rigorous allocation concealment, a computerized random numbers generator (www.randomization.com) was used by an independent statistical expert, who was not involved in the recruitment or clinical assessment, to create the allocation sequence. The enrollment process was managed by the principal investigator, while the concealment mechanism was maintained through the use of sequentially numbered, opaque, sealed envelopes (SNOSE) containing the group assignments. These envelopes were opened only after the participant’s baseline characteristics were recorded and they were formally enrolled in the study.

The randomization process was double-blinded. Specifically, the participants, the clinical investigators responsible for distributing the capsules and monitoring the patients, and the laboratory staff performing the biochemical analyses were all blinded to the group assignments. The capsules were identical in appearance, smell, and packaging to further support the double-blind design. Furthermore, the statistical expert remained blinded during the data processing phase; the allocation code was not disclosed to the analysts until the final database was closed and the statistical analysis was completed.

Throughout the study, we conducted monthly follow-up phone visits to evaluate compliance and adherence to the protocol requirements and monitor any adverse events (AEs). AEs were defined as any untoward medical occurrence in a participant, regardless of its causal relationship with the study intervention. AEs were monitored throughout the study period via participant diaries and physical examinations at each visit. The severity of AEs was graded according to a three-point scale: Mild (transient, easily tolerated, no disruption of normal activities), Moderate (sufficiently discomforting to interfere with normal activities), and Severe (incapacitating, preventing normal activities). If a patient experienced a minor AE, they had the choice to either continue or discontinue using the sachets, but they were still requested to complete follow-up visits. Participants who developed severe diarrhea, nausea/vomiting, or sepsis or who took antibiotics during the study were not invited to the final visit.

To assess patient compliance, the investigator counted the remaining capsules at the end of the treatment and as well cheked the data from the diary. A participant was considered to have good compliance if less than 15% of the capsules remained. Any data from participants who missed more than 15% of the suggested doses were excluded from the final results.

### Supplements

During the study, participants received a twice-daily oral dose of “Del-Immune V^®^ Extra” (containing cell lysate with DNA fragments of the probiotic strain Lactobacillus rhamnosus DV - NRRLB-68023) at a dose of 100 mg or a placebo in capsules. The dietary supplement “Del-Immune V^®^ Extra” in capsules was produced by MirImmunoPharm LLC (Ukraine) in cooperation with Stellar Biotics, LLC (USA). The placebo capsules contained microcrystalline cellulose, which was identical in color, weight, and appearance to the active product capsules and were packaged the same way.

### Outcomes assessment and measurement

After providing informed consent, the patients provided blood serum samples in a fasting state, which were immediately frozen at -20 °C. Relevant clinical and demographic information was collected for each individual. Laboratory studies were conducted in a certified Medical Laboratory CSD, Kyiv, Ukraine.

The main parameters evaluated in the study were glycated HbA1c levels and FPG. FPG was assessed using the enzymatic Trinder method on an Exan analyzer. HbA1c was quantified through an immunoturbidimetric assay using the Cobas 6000 system (Roche Diagnostics, Basel, Switzerland), with a normal reference range of 4.8% to 5.9%. The measurement of HbA1c followed the standardization protocols of the DCCT (Diabetes Control and Complications Trial) and the NGSP (National Glycohemoglobin Standardization Program).

Secondary endpoints included the evaluation of HOMA-IR (homeostatic model assessment of insulin resistance), insulin sensitivity (%S), and β-cell function (%B), as determined via the HOMA-2 model. These indices were calculated using the official software provided by the Oxford Centre for Diabetes, Endocrinology and Metabolism (available at http://www.dtu.ox.ac.uk/homacalculator/index.php). C-peptide concentration was measured using a chemiluminescence immunoassay with commercial kits (Immulite, Siemens AG, Germany), and results were reported in ng/ml.

Anthropometric measurements were carried out for all participants. Body height (BH) was recorded with a precision of 0.001 meters, and body weight (BW) was determined with a precision of 0.001 kilograms using calibrated medical scales. The body mass index (BMI) was computed using the Quetelet formula:


$$\text{BMI }=\text{ BW}/\text{B}{\text{H}}^{2}.$$

The waist circumference (WC) was measured at the level of the belly button using a flexible tape, ensuring a high degree of accuracy of 0.001 m.

### Sample size calculation

The primary outcome measure of the investigation is HbA1c level. The sample size was calculated prior to the trial, based on expected differences in HbA1c. The G*Power 3.1.9.7 package was used to calculate the minimal sample size for the t-test (41). Recent meta-analysis of probiotic interventions in T2D populations have reported effect sizes ranging from 0,81 (SMD) (42). The calculation was performed for α=0.05 and 80% power, clinically significant effect (CE) =1% and variability SD = 1% (Effect Size δ=CE/SD=1). Also, we take into account ongoing war in Ukraine which characterized with high rate of internal displacement and add 20% for attrition (43). Total number of subjects will be greater than min 20 in each group.

### Statistical analysis

Statistical processing of the data was carried out using SPSS software, version 20.0 (SPSS Inc., Chicago, IL, USA), and GraphPad Prism, version 6.0 (GraphPad Software, La Jolla, CA, USA). Quantitative variables are expressed as mean values with standard deviations (M ± SD), while categorical data are shown as percentages. The normality of data distribution was assessed using the Kolmogorov–Smirnov test, supplemented by visual inspection of Q-Q plots and histograms to ensure the reliability of the assumptions, especially given the sample size.

To compare outcomes before and after the intervention within each group, paired t-tests were employed. The primary efficacy endpoints were changed from baseline in HbA1c and FPG at end of treatment (visit 2). To account for multiplicity regarding the two primary endpoints, a hierarchical testing procedure was followed: FPG was tested only if the result for HbA1c reached statistical significance (α=0.05). No formal multiplicity adjustment was applied to the secondary outcomes; therefore, all secondary p-values are nominal, and these results should be interpreted as exploratory rather than confirmatory. For intergroup comparisons post-intervention, repeated-measures analysis of variance (RM-ANOVA) was performed, adjusting for baseline levels and potential confounders.

Data analysis was performed using a per-protocol (PP) approach. Out of the 55 randomized participants, 13 were excluded due to loss to follow-up or protocol violations, such as nonadherence or antibiotic intake, resulting in a final PP cohort of 42 participants (approximately 80% of the original sample).

## Results

### Efficacy

Patient enrollment for the study was conducted from March 1 to June 1, 2023, at the Kyiv City Clinical Endocrinology Center and the Endocrinology Department of Bogomolets National Medical University. A total of 140 individuals with T2D were identified through local electronic medical databases and invited for screening based on study eligibility criteria. Of these, 42 did not meet the inclusion requirements – primarily due to elevated HbA1c levels (>10%) or ongoing therapy with modern antidiabetic agents. An additional 43 individuals declined to participate after being informed about the study’s aims, procedures, and design. As a result, 55 eligible participants were randomized in accordance with the protocol into two arms: a placebo group (n=28) and a group receiving Del-Immune V^®^ Extra (n=27). The enrolled patients’ baseline demographic and clinical characteristics did not significantly differ between groups (**Table 1**).

**Table 1:** Baseline clinical parameters in examined patients (M ± SD or %).

Parameter	Placebo group (n=28)	Del-Immune (n=27)
Age, years	56.18 ± 11.73	56.65 ± 12.49
T2D duration, years	14.00 ± 4.19	12.66 ± 10.40
Metformin, % (n)	57.1 (16)	63.0 (17)
Metformin daily dosage, mg	1362.50 ± 401.04	1350.18 ± 426.46
Sulfonylureas, % (n)	25.0 (7)	29.6 (8)
Insulin human, % (n)	14.3 (4)	14.8 (4)
Insulin analogs, % (n)	35.7 (10)	22.2 (6)
Insulin daily dosage, IU	27.14 ± 8.02	30.30 ± 6.92
Diabetic nephropathy, % (n)	10.7 (3)	11.1 (3)
Diabetic peripheral neuropathy, % (n)	46.4 (13)	40.7 (11)
Diabetic retinopathy, % (n)	46.4 (13)	37.0 (10)

**Figure 1:**
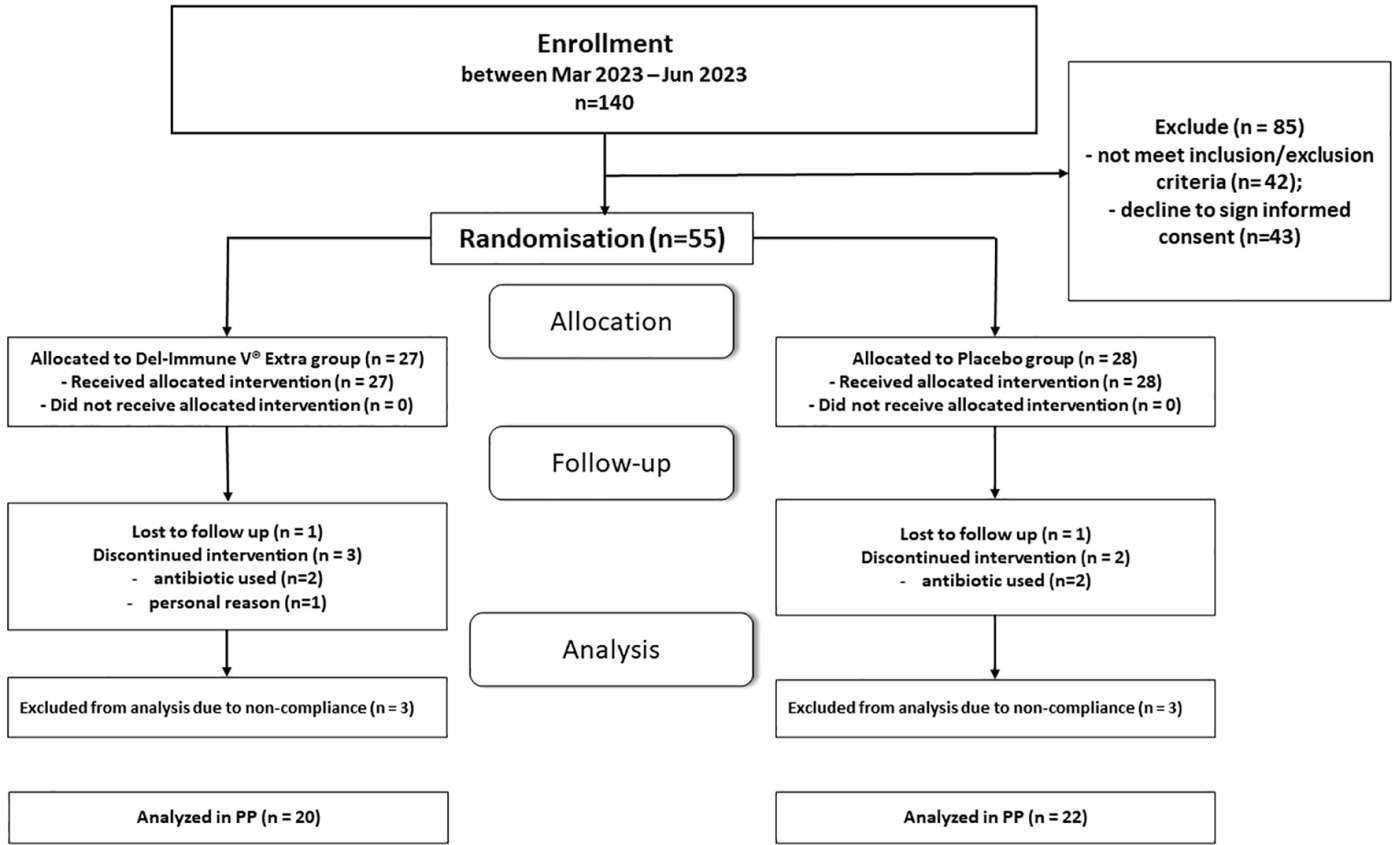
Consolidated standards of reporting trials (CONSORT) flow chart trial protocol.

The PP analysis ultimately included 42 participants (76.4% of the randomized 55): 22 in the placebo group and 20 in the intervention group. This analysis represents a complete-case dataset; notably, no data imputation was performed for missing values. Reasons for exclusion from the per-protocol population are detailed in the CONSORT flowchart (**Figure 1**). All study participants received standard treatment, which consisted of medical guidance, diabetes education, and lifestyle recommendations. Participants did not report any significant changes in their diet or exercise routines. On average, participants in both groups achieved a comparable daily activity level, with mean step counts of 6370.5 ± 813.87 in the intervention group and 6571.72 ± 733.44 in the placebo group (p=0.404). These data provide supportive evidence that physical activity levels were generally balanced across the cohorts during the study period. High adherence to dietary recommendations was observed in more than 85% of participants. Adherence to the assigned intervention was high, with more than 90% of the prescribed sachets consumed in the double-blind phase. The products were generally well accepted in terms of taste and appearance, with good overall tolerability.

**Figure 2:**
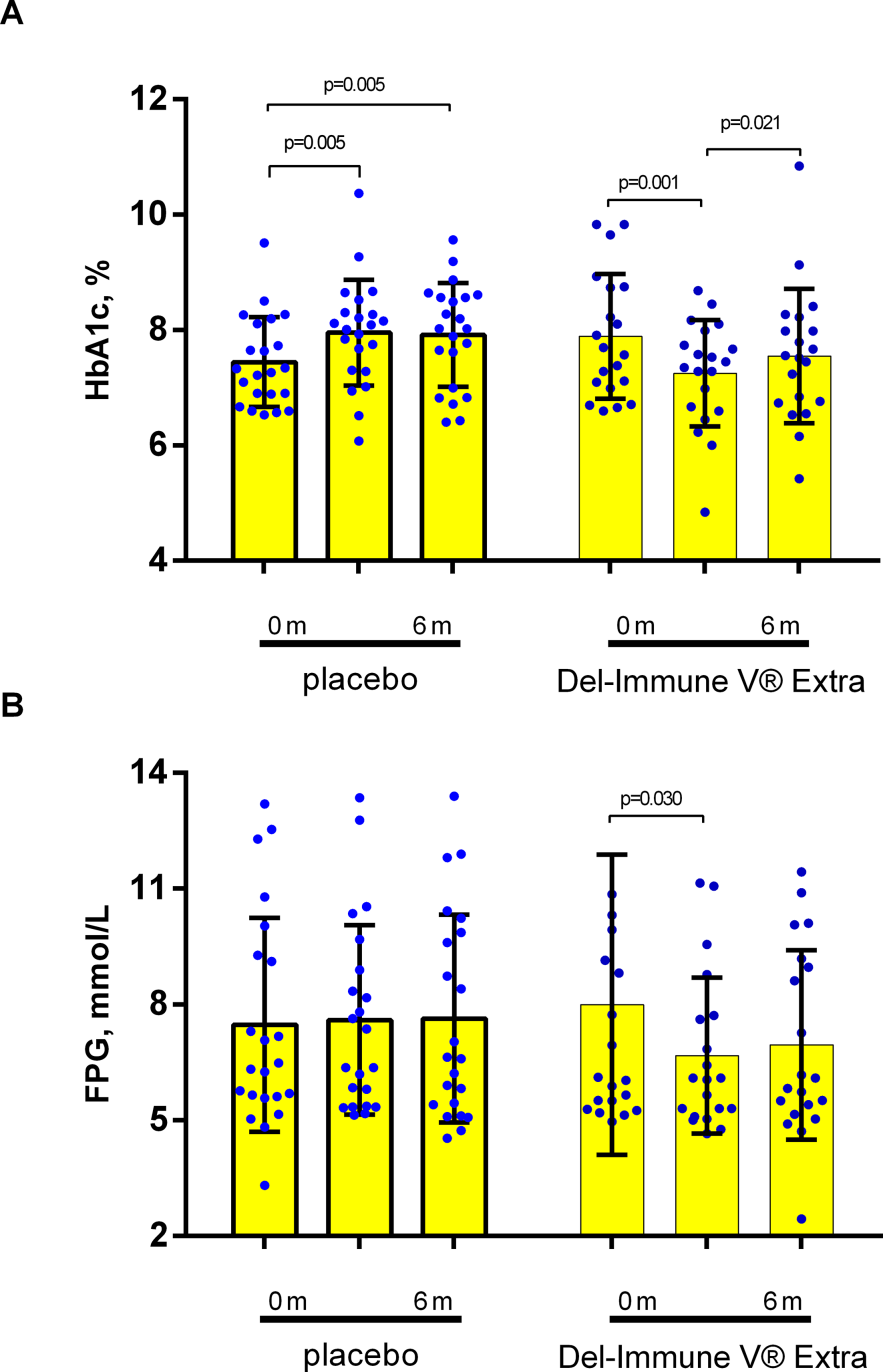
Primary outcomes analysis with accent on glycemic-related parameter changes [**(A)** HbA1c; **(B)** FPG]. Data presented as M ± SD.

The primary analysis using repeated-measures ANOVA demonstrated a highly significant overall treatment effect for HbA1c (p<0.001), indicating distinct patterns of glycemic changes between the groups. After 3 months of active treatment, the Del-Immune group showed a mean absolute reduction in HbA1c of –0.63% (95% CI: –0.98 to –0.28), whereas the placebo group experienced a significant increase of 0.50% (95% CI: 0.17 to 0.84). At the 6-month follow-up visit, the reduction in the bacterial lysate group remained lower than baseline (–0.33%; 95% CI: –0.69 to 0.02), while the placebo group maintained an elevated level (0.47%; 95% CI: 0.15 to 0.78) (**Table 2**). For FPG, a positive treatment trend was observed (p=0.086), with the intervention group showing a sustained decrease compared to the stable or increasing levels in the placebo group (**Table 2**). The intragroup changes of primary endpoints presented at **Figure 2**.

**Table 2:** Intergroup analysis of primary outcomes.

Parameters	Baseline	Mean absolute changes from baseline		P	
		Δ 3 month	Δ 6 month	Time effect	Time*treatment effect
HbA1c, %				0.313	<0.001
Placebo	7.44 ± 0.77	0.50 (0.17 to 0.84)	0.47 (0.15 to 0.78)		
Del-Immune	7.88 ± 1.07	-0.63 (-0.98 to -0.28)	-0.33 (-0.69 to 0.02)		
FPG, mmol/L				0.168	0.086
Placebo	7.47 ± 2.77	0.12 (-0.51 to 0.75)	0.15 (-0.61 to 0.92)		
Del-Immune	7.99 ± 3.89	-1.32 (-2.49 to -0.14)	-1.04 (-2.33 to 0.24)		

Baseline values presented as M ± SD. The mean absolute values were calculated as post-treatment minus baseline and presented as mean differences and 95% confidence intervals (CI). p “time” - measures the effect of time across all three visits for both groups combined; p (time*treatment) evaluates if the intervention group showed a different pattern of change compared to the placebo group during both the active treatment (0–3 months) and the subsequent follow-up (3–6 months).

**Figure 3:**
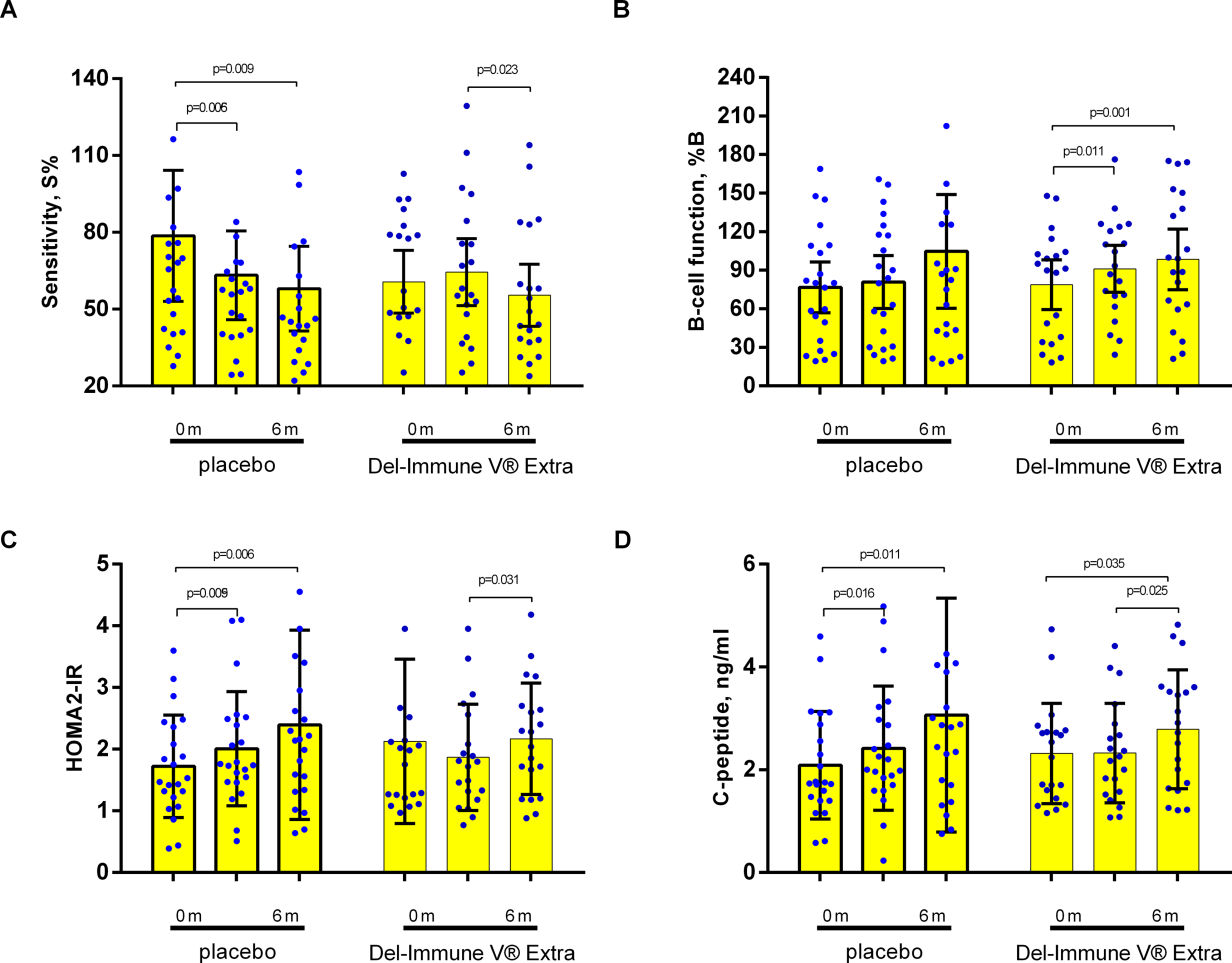
Secondary outcomes analysis with accent on HOMA-2 model parameters [**(A)** insulin sensitivity (% S); **(B)** β-cell functional activity - % B; **(C)** HOMA-2IR; **(D)** C-peptide]. Data presented as M ± SD.

The exploratory intergroup analysis of secondary outcomes revealed a significant treatment effect for WC and insulin sensitivity (%S) (**Table 3**). For WC, while both groups showed a numerical reduction after 3 months, a significant mean reduction was observed only in the Del-Immune group (–1.55 cm; 95% CI: –2.31 to –0.78; p=0.021 for intergroup change), which remained below baseline at follow-up. In contrast, the placebo group showed no significant changes over time. Regarding insulin sensitivity (%S), the intergroup analysis demonstrated significant multidirectional changes, driven by an increase in the Del-Immune group (3.76; 95% CI –2.65 to 10.17) compared to a decrease (–15.42; 95% CI –26.02 to –4.83) in the placebo group (p=0.013). The Del-Immune group demonstrated a baseline-to-follow-up divergence compared to the placebo group, where parameters tended to worsen at both 3 and 6 months (**Figure 3A**).

**Table 3:** Intergroup analysis of secondary outcomes.

Parameters	Baseline	Mean absolute changes from baseline		p	
		Δ 3 month	Δ 6 month	Time effect	Time*treatment effect
Sensitivity, %S				0.016	0.013
Placebo	78.63 ± 57.79	-15.42 (-26.02 to -4.83)	-20.66 (-35.63 to 5.69)		
Del-Immune	60.69 ± 26.09	3.76 (-2.65 to 10.17)	-5.29 (-13.55 to 2.97)		
%B				0.020	0.448
Placebo	76.67 ± 44.51	4.23 (-10.02 to 18.49)	28.03 (-5.12 to 61.19)		
Del-Immune	78.77 ± 41.26	12.4 (3.14 to 21.66)	19.75 (9.46 to 30.04)		
HOMA-2IR				0.046	0.062
Placebo	1.72 ± 0.82	0.28 (0.07 to 0.48)	0.67 (0.21 to 1.12)		
Del-Immune	2.12 ± 1.33	-0.25 (-0.69 to 0.18)	0.04 (-0.47 to 0.56)		
C-peptide, ng/mL				0.004	0.209
Placebo	2.11 ± 1.06	0.33 (0.06 to 0.59)	0.90 (0.22 to 1.57)		
Del-Immune	2.31 ± 0.97	0.01 (-0.32 to 0.33)	0.46 (0.03 to 1.90)		
BMI, kg/m^2^				0.001	0.456
Placebo	31.97 ± 3.65	-0.38 (-0.81 to 0.05)	-0.11 (-0.71 to 0.48)		
Del-Immune	32.89 ± 3.87	-0.93 (-1.59 to -0.26)	-0.68 (-1.32 to -0.04)		
Weight, kg				0.004	0.245
Placebo	92.07 ± 15.21	-0.77 (-1.91 to 0.37)	-0.19 (-1.74 to 1.36)		
Del-Immune	94.47 ± 13.49	-2.44 (-4.15 to -0.72)	-1.93 (-3.69 to -0.16)		
WC, cm				<0.001	0.021
Placebo	103.95 ± 10.86	-0.22 (-0.83 to 0.38)	0.22 (-0.55 to 1.01)		
Del-Immune	107.4 ± 10.35	-1.55 (-2.31 to -0.78)	-1.1 (-1.68 to -0.51)		

Baseline values presented as M ± SD. The mean absolute values were calculated as post-treatment minus baseline and presented as mean differences and 95% confidence intervals (CI). p “time” - measures the effect of time across all three visits for both groups combined; p (time*treatment) evaluates if the intervention group showed a different pattern of change compared to the placebo group during both the active treatment (0–3 months) and the subsequent follow-up (3–6 months).

**Figure 4:**
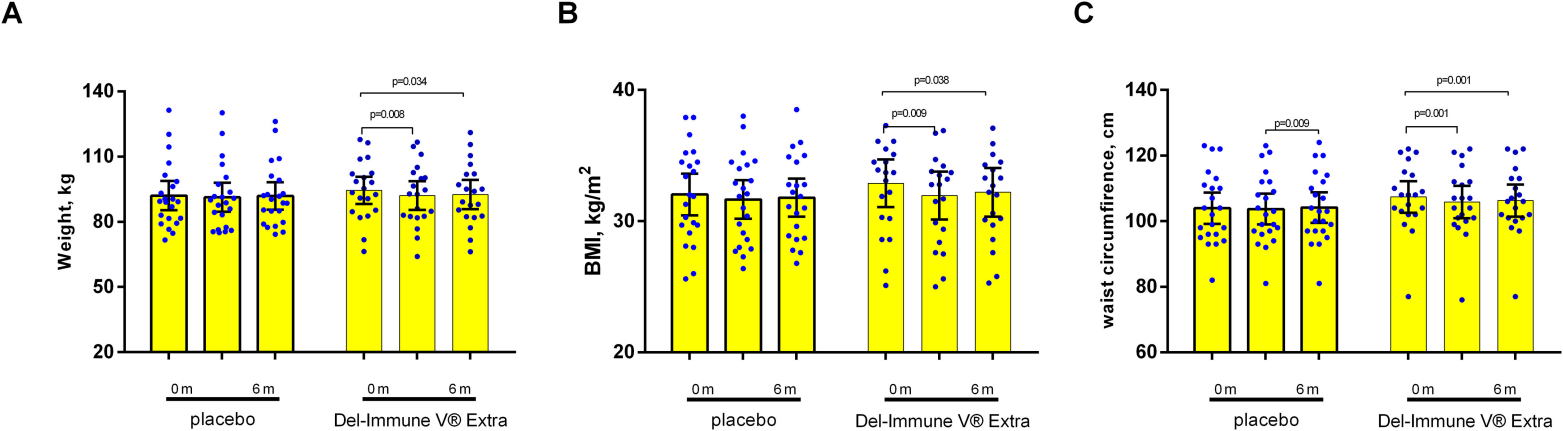
Secondary outcomes analysis with accent on anthropometric parameters [**(A)** body weight; **(B)** body mass index; **(C)** waist circumference]. Data presented as M ± SD.

Other secondary metabolic and anthropometric parameters, including β-cell function (%B), HOMA-2IR, C-peptide, and BMI, did not show statistically significant between-group differences (p>0.05 for all treatment effects). Notably, while within-group improvements in %B (**Figure 3**) and BMI (**Figure 4**) were observed in the Del-Immune group (p<0.05), these did not reach statistical significance when compared directly to the placebo group. As no formal multiplicity adjustment was applied to these secondary analyses, all reported p-values are nominal, and these findings should be interpreted as hypothesis-generating.

### Safety

The total incidence of adverse events (AEs) did not differ between groups at the end of treatment period. The most common AE was mild gastrointestinal (GI) discomfort (placebo: 5 patients - 17.9% vs Del-Immune group: 4 patients - 14.8%; p=0.760) All events were transitory and resolved spontaneously. Metformin is known to cause GI symptoms. In our study the frequency of GI symptoms were similar in metformin recipients (2 participants in each group, 7.4 vs 7.2%).

The main reported AEs were gastrointestinal complaints, which were mild in their intensity. The most frequent complain was constipation, which were observed in 2 patients that received bacterial lysate derived from *L. rhamnosus* DV (7.4%) and 1 from placebo group (3.6%; p=0.531). One patient from each group experienced heartburn and transient diarrhea (p=0.979). Additionally, one participant that received placebo reported short-term abdominal pain with bloating.

During the follow-up period, patients didn’t report any AE. Also, no severe adverse events were reported during RCT.

## Discussion

In this double-blind, placebo-controlled RCT, adjunctive supplementation with a bacterial lysate derived from *L. rhamnosus* DV resulted in a significant between-group reduction in HbA1c compared with placebo after three months of intervention, while a trend toward improvement in FPG was observed. Improvements in anthropometric parameters were also observed, with a statistically significant between-group effect detected for WC. In line with the hierarchical testing procedure, secondary outcomes were interpreted as exploratory due to the nominal nature of their p-values. These findings suggest that supplementation with bacterial lysate–derived postbiotic components may improve metabolic control when added to standard therapy in individuals with T2D.

In gut dysbiosis associated with T2D, microbiota increases the presence of opportunistic pro-inflammatory bacteria, leading to local intestinal inflammation. This results in heightened intestinal permeability, often referred to as “leaky gut.” As a consequence, pathogen-associated molecular patterns (PAMPs) – pro-inflammatory substances from bacterial cell walls such as peptidoglycans and lipopolysaccharides – can more easily enter the bloodstream, activating vascular endothelial and peripheral immune cells, which indicates systemic inflammation (44, 45). This inflammation is a key factor in metabolic syndrome, increasing the risks of hypertension, visceral obesity, and dyslipidemia, which can damage pancreatic β-cells and reduce insulin secretion, thus contributing to T2D (46, 47).

However, complete therapeutic probiotic potential in the food and pharmaceutical industries has been limited due to multiple technological and biological challenges, such as ensuring probiotic viability and efficacy during the manufacturing process and consumption by individual customers.

As a result, there is a growing shift away from focusing solely on the viability and quantity of probiotic cells towards nonviable bacterial-derived biomolecules, known as postbiotics and metabiotics. Biologically active metabolites and bacterial cell fragments are emerging concepts in functional foods and biomedicine, as they provide a wide range of health-promoting properties (23, 48).

Studies in animal models have demonstrated the effects of nonliving bacteria, offering advantages over live bacteria. For example, postbiotics derived from *Limosilactobacillus fermentum* and *L. delbrueckii* influence mouse behavior (49). Compared with control animals, treated animals presented increased sociability, lower stress hormone levels, and changes in gut microbiota composition (49).

Postbiotic substances have shown promise in clinical interventions. In a recent clinical study, researchers investigated the effects of supplementation with a postbiotic extract of *B. breve* BB091109 on proinflammatory cytokine levels and markers of endocrine function. The results showed that taking *B. breve* postbiotic extract for 90 days significantly reduced CRP and IL-6 levels and affected dehydroepiandrosterone (DHEA), estradiol, and estriol. In conclusion, the supplement improved endocrine function in women over 40 years of age and resulted in positive changes in inflammatory markers. These findings suggest that this supplement could be beneficial for promoting hormonal balance and reducing inflammation in this age group (29). We found these studies intriguing because the postbiotic demonstrated a potent anti-inflammatory effect. T2D is characterized by chronic low-grade inflammation. It is thought that increased inflammation significantly impacts glucose metabolism. Chronic systemic inflammation leads to oxidative stress, causing β-cell dysfunction and the expansion of α-cells in the pancreas, ultimately contributing to the progression of T2D in individuals with obesity (50–53). The difficulty of achieving optimal glycemic control highlights the need to explore additional or alternative pathways to offer more therapeutic strategies for treating T2D. Specifically, we investigated whether probiotic derivatives, i.e., postbiotic metabolites and structural fragments of metabiotic cells, would have a positive effect on people with T2D.

In this study, we examined the effectiveness and safety of an innovative product containing cell lysate and DNA fragments from the probiotic strain *L. rhamnosus* DV-NRRLB-68023 as an additional treatment for people with T2D. The oral dose was 100 mg twice daily compared with a placebo in capsules. After three months of treatment with bacterial lysate, there was a significant decrease in HbA1c. The observed improvement in glycemic control was accompanied by changes in insulin sensitivity (%S); however, as direct measurements were not performed, these findings should be interpreted with caution. We acknowledge that the concurrent use of multiple antidiabetic medications in patients with T2D could have influenced glycemic outcomes; however, the dosages and regimens remained stable during the RCT.

The observed reduction in HbA1c of approximately 0.6% is comparable to the magnitude reported in several trials evaluating adjunctive nutritional and microbiota-targeted interventions and may be considered clinically meaningful in the context of combination therapy. The effect size observed in the present study is smaller than that typically reported with first-line pharmacological agents such as metformin or GLP-1 receptor agonists, which generally reduce HbA1c by approximately 1–1.5%, but is consistent with the expected contribution of adjunctive non-pharmacological interventions (54, 55).

Anthropometric parameters decreased (only WC significantly in between-group) in the group receiving bacterial lysate derived from *L. rhamnosus* DV. We noted that the endpoints worsened during follow-up visits compared with those at the end of the treatment visit. Importantly, the parameters did not return to their original baseline levels. All participants received standardized dietary and physical activity recommendations before randomization. Although these measures were necessary to ensure comparable baseline lifestyle conditions, they may have also contributed to metabolic improvements within the groups. Therefore, the interpretation of treatment efficacy should focus primarily on differences between groups rather than changes within each group. It’s important to note that a balanced diet was prescribed, with an emphasis on dietary fiber to help modulate the gut microbiota. Individual variations in macronutrient composition may have influenced the treatment effects, which is a limitation of this study.

These results align with a growing body of literature emphasizing the potential benefits of probiotics and symbiotics in treating diabetes, especially in improving glycemic control (56–58). A meta-analysis revealed significant improvements in three important diabetes indicators: HbA1c, FPG, and serum insulin levels (59). These findings add to our current knowledge about the potential of probiotics and symbionts in the treatment of diabetes. However, the variability in results indicates that effectiveness may depend on various factors, including the type and severity of diabetes, as well as the specific strains of probiotics or symbiotics used (59). In our previous RCT (11), we reported that taking a live multistrain probiotic for 2 months improved β-cell function and reduced FPG and HbA1c levels. Probiotic therapy also reduced proinflammatory cytokine levels, indicating a positive impact on chronic systemic inflammation in T2D patients (60). Based on our findings, we concluded that this therapy is safe and has minimal side effects. It has the potential to regulate glucose metabolism, improve β-cell function, and reduce chronic inflammation in individuals with T2D. Thus, in the present study, we demonstrated that the cell lysate and DNA fragments of the probiotic strain *L. rhamnosus* DV act similarly to those of the multistrain probiotic we previously used widely in clinical practice (11, 61). So, lysates and DNA fragments (postbiotics) act through pathways similar to those of live probiotics, modulating the immune system and reducing inflammation. Their main advantages are enhanced stability, absence of infection risk, and suitability for immunocompromised individuals.

It is crucial to differentiate the current intervention from traditional strain-specific probiotic supplementation using live *L. rhamnosus*. Numerous randomized clinical trials assessing viable *L. rhamnosus* preparations in metabolic disorders have reported mixed or neutral effects on glycemic outcomes (59, 62–64). In some cases, there were no significant differences in HbA1c levels between groups. For instance, a study that supplemented individuals with T2D with *Lactobacillus rhamnosus GG* for eight weeks found no significant improvement in HbA1c, despite alterations in the expression of genes related to the intestinal barrier (62).

In contrast, the intervention examined in this study involves a lyophilized enzymatic bacterial lysate that contains structural postbiotic components, including muramyl peptides and bacterial DNA fragments (23). These components may directly interact with host innate immune receptors, such as NOD-like and Toll-like receptors (23, 65–68). As a result, they may exert immunometabolic effects independent of bacterial viability, the efficiency of intestinal colonization, or the baseline composition of the microbiota. This mechanistic distinction may help explain the differences observed between the outcomes of live probiotic supplementation and those of bacterial lysate–based postbiotic preparations.

In this study, we also examined the effects of discontinuing the testing supplement. After stopping the intervention, we observed that health indicators worsened during follow-up visits compared to their status at the end of the treatment period. However, these indicators did not return to their original baseline levels. This suggests that postbiotics may have only temporary effects; the duration of exposure might be too short to induce lasting changes in gut microbiome composition, resulting in effects that are not sustainable. When prescribing postbiotics, it is important to consider that postbiotics are transient and their effects diminish once administration ceases, as they do not permanently colonize the gut microbiome.

Research on the metabolic effects of postbiotic preparations derived from bacterial lysates in individuals with T2D is currently limited. This study provides additional clinical evidence supporting their potential as supplementary metabolic interventions. Specifically, it suggests that adding probiotic lysate to standard antidiabetic therapy can improve glycemic-related factors and enhance insulin sensitivity in individuals with T2D. It should be noted that participants receiving SGLT-2 inhibitors, GLP-1 receptor agonists, DPP-4 inhibitors, or pioglitazone were excluded to minimize confounding effects related to their independent metabolic and weight-modifying actions. While this approach improved internal validity, it may limit generalizability of the findings to patients receiving contemporary multidrug treatment regimens.

Our study has several limitations that should be taken into account. First, the analysis was restricted to the PP population, and the lack of an ITT analysis may limit the generalizability of the findings and potentially overestimate treatment effects. Furthermore, the relatively small sample size restricted statistical power and subgroup evaluations, and no formal multiplicity adjustment was applied to secondary outcomes. Second, participant attrition during follow-up might introduce potential bias. Third, while lifestyle recommendations were standardized, the inherent variability of self-reported dietary intake and physical activity in an outpatient setting remains a limitation, as these factors could have influenced the observed metabolic outcomes. Additionally, we did not assess important mechanistic measurements, such as gut microbiota composition, inflammatory biomarkers, and markers of intestinal permeability. This absence restricts our understanding of the biological pathways that could explain the observed effects. Lastly, the relatively short duration of supplementation prevents us from drawing conclusions about the long-term sustainability of the intervention’s metabolic benefits.

Despite these limitations, there is growing interest in using probiotic lysates (postbiotics and metabiotics) to modulate inflammatory processes and the gut microbiota, with the goal of improving host metabolism. However, further evidence from human trials is needed to confirm these effects in individuals with T2D and to conduct a meta-analysis.

## Conclusion

The adjunctive administration of probiotic lysate, which contains postbiotic metabolites and metabiotic cell fragments, to people with T2D has been shown to have beneficial effects on health status. Three-month supplementation with *L. rhamnosus DV* lysate significantly improves glycemic control (HbA1c), improves insulin sensitivity and reduces WC compared to placebo in patients with T2D. However, we observed that these metabolic improvements tended to worsen during the 3-month follow-up period after treatment discontinuation, suggesting that the beneficial effects of the bacterial lysate may require sustained administration or integration with long-term lifestyle interventions to maintain stable glycemic control.

## Data Availability

The raw data supporting the conclusions of this article will be made available by the authors, without undue reservation.
